# Evaluating the Amyloidosis Speakers Bureau: the influence of amyloidosis patients’ narratives on medical students’ knowledge, attitudes, and behavioral intent

**DOI:** 10.12688/mep.19631.1

**Published:** 2023-06-28

**Authors:** Adebanke L. Adebayo, Katherine E. Rowan, Vaishali Sanchorawala, Mackenzie N. Boedicker, Deborah D. Boedicker

**Affiliations:** 1 Department of Communication Studies, Washburn University, Topeka, Kansas, 66621, USA; 2 Department of Communication, George Mason University, Fairfix, Virginia, 22030, USA; 3 Amyloidosis Center, Chobanian and Avedisian School of Medicine, Boston University, Boston, Massachusetts, 02115, USA; 4 Amyloidosis Speakers Bureau / Mackenzie's Mission, Great Falls, Virginia, 22066, USA; 5 Bouvé College of Health Sciences, Northeastern University, Boston, Massachusetts, 02115, USA

**Keywords:** Amyloidosis, Amyloidosis Speakers Bureau (ASB), narratives, patient-centered communication, patient educators

## Abstract

**Background: ** Amyloidosis is a complex multi-systemic disease. Lack of knowledge about amyloidosis and subsequent mis- or under-diagnosis are major obstacles to treatment, which result in life-threatening organ damage, morbidity, and mortality. Hence, the purpose of this study is to explore the effectiveness of amyloidosis patients’ narratives on medical students.

**Methods: ** The Amyloidosis Speakers Bureau (ASB) arranges for amyloidosis patients to speak about their diagnostic and treatment experiences with medical students. Using a randomized post-test only experiment, we compared the effectiveness of patients’ narratives between two groups (treatment and control). Outcome measures included medical students’ intent to actively communicate with patients, acquire knowledge about amyloidosis, and reconsider diagnoses when warranted.

**Results:  **The treatment group (those who listened to an ASB patient speaker) had higher mean differences on all measures, including the desire to improve communication with patients, acquire and apply knowledge of amyloidosis, and willingness to reconsider diagnoses when symptoms are puzzling.

**Conclusions:  **ASB patient educators widened awareness of an under-diagnosed disease. Listening to a patient’s narrative was associated with positive attitudes toward communication with patients, interest in acquiring and applying knowledge of amyloidosis, and humility about diagnosis. Narrative and persuasion theory are used to explain this quantitative evidence of the power of patient narratives.

## Introduction

Amyloidosis is a complex set of diseases caused by misfolded proteins that damage organs ( Dasari *et al.*, 2020). Lack of knowledge about this disease and subsequent mis- or under-diagnosis are major obstacles to treatment ( Dispenzieri & Merlini, 2020; Grogan *et al.*, 2017), which result in life-threatening organ damage, morbidity, and mortality ( Gillmore *et al.*, 2021; McCausland *et al.*, 2018). Amyloidosis presents in many ways, with seemingly unrelated symptoms often associated with more common diseases, making diagnosis extremely difficult ( McCausland *et al.*, 2018). According to the Amyloidosis Research Consortium in 2021, a plurality of patients received at least one misdiagnosis before being correctly diagnosed and multiple patient respondents reported visiting four or more physicians before receiving a diagnosis ( Lousada *et al.*, 2015). There are 30,000 to 45,000 patients living with one manifestation of amyloidosis, AL amyloidosis, in the United States and the European Union ( Lousada *et al.*, 2015).

With these alarming diagnostic challenges and associated health outcomes, delayed diagnosis remains a major obstacle to effective therapy prior to end-stage organ failure. Hence, decreasing the time to diagnosis is essential for the optimal health and survival of amyloidosis patients ( Dispenzieri & Merlini, 2020; McCausland *et al.*, 2018). Without decreasing these delays, recent advances in the treatment of amyloidosis will be under-utilized. Early detection is vital to preventing adverse health outcomes, futile chemotherapy from misdiagnosis and under-diagnosis, waste of time and resources, as well as physical and mental stress on patients related to misdiagnosis.

A key factor to aid early detection is knowledge of the disease. Therefore, the purpose of this study is to learn whether listening to amyloidosis patients’ narratives about their diagnostic and treatment experiences increases medical students’ intent to actively listen to patients, learn about amyloidosis, and revisit initial diagnoses when complex symptoms warrant.

## Literature review

Engel (1977) wrote that to understand patients and give them a sense of being understood, physicians should adopt a biopsychosocial model of care. That is, rather than ignoring patients’ subjective experiences, physicians should develop empathic curiosity about these experiences ( Borrell-Carrio *et al.*, 2004).

There has been an increasing effort to enact the biopsychosocial model of healthcare. One approach is to involve patients in medical education. Reviews find medical students benefit from interaction with patients but that theoretical accounts of how and why they benefit are under-developed ( Gordon *et al.*, 2020). Patients’ narratives are one technique for encouraging empathic curiosity concerning patients’ subjective experiences as well as encouraging learning about diseases that present in complex ways like amyloidosis. It is time for modern medicine to integrate the compassion and empathy gleaned from patients’ narratives to assist in diagnosis and alleviate patients’ suffering and pain ( Langer & Ribarich, 2008).

### Why narrative

Studies that compare and contrast patient narratives versus expository accounts of the same medical information suggest why they influence attitudes important in communication and diagnosis. Snow *et al.* (2016) explored the effect of two videos on medical students’ attitudes toward communicating with patients. In this study, 135 students were assigned randomly either to the experimental group, watching a video where a patient described a surgical procedure on the cervix, or the control group, who experienced a doctor’s description. Each video described steps to remove precancerous lesions. The doctor described major steps in the procedure. In contrast, the patient described how it felt: "It’s basically they pass a hot wire loop through your cervix to remove abnormal cells. It does sound quite scary but it’s not. You actually don’t feel it because you get anesthetized…I think the worst thing is having your legs up on the stirrups” (p. 1230). Snow and associates found the experimental group had more confidence that they could respond to a patient’s concerns about the procedure than did the control. Listening to the patient prepared them for what a patient might ask.

Listening to the patient narratives allows for the humanization of care and not just a list of general attributes and characteristics ( Todres *et al.*, 2009). Hence, instead of viewing themselves as passive recipients of illness and treatment, patients gain a sense of participating in the healing process ( Keshet *et al.*, 2015).

Another study illuminated narrative’s power to educate and motivate health information seeking. Adebayo and colleagues (2020) studied the impact of narrative versus expository text (a summary of information) provided to pregnant women in physicians’ waiting rooms. Women who experienced a narrative account of climate change were more likely to use an app to learn about high heat and humidity and its potential to harm them than were those who read summaries. Similarly, Borah and colleagues (2022) in their study to examine the influence of narrative vs statistical messages on COVID-19 related information seeking and COVID-19 vaccine intention found that narrative messages were more persuasive for education and information seeking behaviors.

*Impact on attitudes relevant to diagnosis.* Narratives presenting a patient case are common in medical journals, textbooks, and lectures. Less common is having a patient who has a rare disease speak directly to medical students. Research on “patient educators” analyzes this instructional approach. Edwards *et al.* (2016) examined listening to a patient’s presentation on pemphigus vulgaris, an autoimmune disease that causes blisters on skin and mucous membranes in the mouth and other body cavities. A patient, selected by faculty, presented her diagnostic and treatment experience at two universities. Dentistry students reported that it was motivating to listen to an actual patient and that they were more likely to remember pemphigus vulgaris after listening to her than had they read a textbook account of this disease.

*Challenges*. A single case or narrative has advantages and limitations. On the one hand, experts in risk analysis can consider both vivid examples and trend data, without having the vivid example eclipse understanding of the trend ( Dieckmann *et al.* 2009). On the other hand, in a systematic review of internet-based patient narratives, Drewniak and colleagues (2020) found limited evidence of beneficial effects from patient narratives. However, their study found that patient narratives are promising means to support users in improving their understanding of certain health conditions to possibly provide emotional support and have an impact on behavioral changes. They note that narratives vary and are hard to compare with one another, which makes quantitative assessment of their impacts challenging.

### Effects of narrative

In contrast to Drewniak and colleagues’ conclusions about the limited effects of patient narratives, others find narrative used in health contexts has frequent, predictable effects. Slater and Rouner (2002) draw from Petty and Cacioppo’s Elaboration Likelihood Model (1986) to compare the effects of narrative with expository discourse. They and others ( Shaffer *et al.*, 2018) report that narrative is easier to understand than textbook or lecture material, more apt to lead to durable memories than expository discourse, and more persuasive.

***Easy to comprehend.*** Human beings begin listening to narratives as infants. According to Fisher, humans are *homo narrans–*storytellers (1984). Psychologists studying prose learning find that narratives are easily remembered ( Bower *et al.*, 1979), and, as Slater and Rounder (2002) write (p. 179), there is an “automaticity” associated with processing narrative, or stories, in contrast to the more effortful processing required to learn from texts or lectures about a disease. The familiarity and simplicity of narratives may help create memories of an unfamiliar disease such as amyloidosis, more so than an expository presentation. Several studies have found that narratives are easier to comprehend than “traditional scientific” forms of communication ( Adebayo *et al*., 2020; Green, 2006; Hinyard & Kreuter, 2007), because narrative and traditional scientific messages have two distinct cognitive processes. The narrative pathway is situation-based and associated with increase recall, easy comprehension, and shorter reading times.

***Persuasive.*** Shaffer and colleagues (2018) report narrative, as compared to expository discourse, is less likely to elicit counter-arguing. According to Slater and Rouner (2002), narratives engender involvement and empathy with characters. The stories amyloidosis patients share often emphasize the confusing nature of symptoms and the number of doctors they visit prior to being diagnosed ( Lousada *et al.*, 2015). Learning about these difficulties through a patient’s narrative might elicit empathy from medical students and may reduce counter-arguing from those resistant to viewing patients as partners in disease management.

In sum, narratives can be memorable, persuasive, and less likely than expository discourse to encourage disagreement. This suggests that the dozens of patient educators who speak to medical students through the ASB will create durable memories of amyloidosis and influence attitudes about listening to patients. Because the ASB has provided dozens of patient educators at dozens of medical schools since 2019, these conditions create an opportunity to study the effects of patient narratives on medical students quantitatively and theoretically. We, therefore, offer these hypotheses:

H1: Listening to an amyloidosis patient from the ASB will increase medical students’:

H1a: Intent to actively listen to patients’ thoughts, feelings, and information.
H1b: Likelihood of considering amyloidosis when they observe clusters of symptoms that do not fit one disease.
H1c: Willingness to reconsider initial diagnoses when symptoms are puzzling.

## Methods

### Procedures

This study conducted a randomized experiment with two groups, using a post-test measurement only. To mitigate sensitizing the study participants, the possibility of conducting pretest measures was eliminated. Hence, the group that was not exposed to the treatment was the control group. The control group completed the survey before being exposed to a patient presentation (treatment), while the treatment group completed the survey after listening to a patient presentation.

To thank medical student group organizers for hosting the ASB and inviting medical students to attend, the ASB provided each a $25 eCard. In addition, up to four attendees earned a chance to receive a $25 eCard, distributed randomly to those who completed the survey.

Prior to participating, medical students were invited to take part in this study and informed about all study procedures, as well as their rights as participants. Those who consented did so electronically by clicking a button on an online document. This protocol and all study procedures were approved by the (name redacted) university institutional review board.

### Treatment

The ASB patient presentation is a 30-minute presentation that teaches this disease through the patient’s experience. Each presentation covered symptoms, diagnosis, treatment, caregiver support, and resources. Other topics, such as the importance of patient advocacy for themselves and the value of effective patient-physician communication are also discussed. In contrast to presentations typical in textbooks and lectures, ASB patient educators make their presentations personal. That is, ASB patient educators share their feelings of hope, fear, and uncertainty in their narratives (see Appendix B, *Extended data* for speaker story development guide [ Adebayo, 2023b]). This approach increases the likelihood that patients’ narratives are memorable and likely to motivate medical students to value learning about patients’ experiences, about amyloidosis, and the importance of humility concerning diagnoses.

### Measures

Survey items were developed with input from the study’s physician co-author who treats amyloidosis patients and studies this disease. Items were also generated by following Ajzen’s Theory of Planned Behavior (2011), which describes attitudes that predict behavioral intentions, such as intent to communicate in a certain way with patients or intent to learn about a disease.

Survey items were measured on a 7-point Likert scale using both unlikely to likely response options and strongly disagree to strongly agree options. For three outcome measures with more than one question, the answers to each question were combined in a simple additive scale. All measures used in this study were developed and pilot tested by the investigators for this study (see Appendix A, *Extended data* for survey measures [ Adebayo, 2023b]).

***Active communication with patients.*** Increasingly, medical practice and medical education encourage “active communication,” from physicians, where physicians view it as their responsibility to create physician-patient encounters where patients feel encouraged to share information, express their concerns, and share in decision-making (e.g., Baker *et al.*, 2021). These attitudes assist diagnosis and treatment, especially in the case of under-diagnosed diseases. This outcome was assessed using three items. For example, “I intend to improve my communication skills related to hard-to-diagnose health issues such as amyloidosis.”

***Intention to acquire and apply knowledge of amyloidosis.*** Accurate and timely diagnosis is more possible when there is awareness of amyloidosis as a possible diagnosis and openness to learning about patients’ subjective experiences. This outcome was assessed using three items. For example, “When observing a constellation of clinical syndromes that do not fit one disease, how likely are you to think of amyloidosis?”

***Willingness to reconsider diagnoses.*** Many are reluctant to admit “wrongness,” defined as past statements later shown to be wrong ( Fetterman *et al.*, 2019). This reluctance may come from fear of seeming incompetent, though evidence suggests those who admit wrong publicly may be viewed as *more* competent as well as agreeable ( Fetterman *et al.*, 2019). A further challenge in physician-patient relationships is that medical care may harm patients ( Gigerenzer *et al.*, 2007). Diseases like amyloidosis, where symptoms do not fit common ailments and affect multiple organs, are difficult for any physician to treat. Therefore, willingness to reconsider diagnoses when compelling information warrants is essential. This outcome was assessed using two items. For example, “I believe that the combined knowledge of the patient and physician can help provide relevant information about symptoms and aid easier diagnosis.”

### Participants

This study was conducted in 32 U.S. medical schools with a total of 361 participants from which the treatment (n = 108) and control group (n = 108) were randomly selected. This study includes 216 participants. The mean age of participants was 25.12 years ( *SD* = 3.81); 65.3% ( *n* = 141) of participants were female and 34.7% ( *n* = 75) were male. A majority were White (52.8%, *n* = 114); the others were Asian (27.8%, *n* = 60), Black/African American (6.9%, *n* = 15), Hispanic/Latino (6.5%, *n* = 14), Native Hawaiian or Pacific Islander (0.5%, *n* = 1), and “other” (5.6%, *n* = 12). A majority reported learning about amyloidosis in medical school (69.9%, *n* = 151). A plurality were first year students (36.6%, *n* = 79); a third were second year (33.8%, *n* = 73); a fifth were third year (20.4%, *n* = 44), and the remainder fourth year (9.3%, *n* = 20).

### Patient educators

In this study, there were 40 patient educator presentations with 23 unique patient educators (most of the patient educators only spoke once). Most of the patient educators were female 52% ( *n* =12) and 48% ( *n* = 11) were male. At these 32 medical schools there were 40 patient educator presentations, where five medical schools had more than one patient educator; two schools had two speakers; and three schools had three speakers. Although there was a template for the patient presentation (see Appendix B, *Extended data* [ Adebayo, 2023b]), the patient educators had differing types of amyloidosis, affecting differing sets of organs including but not limited to the heart, kidney, liver, GI tract, neuropathy, carpal tunnel syndrome, spinal stenosis, bicep bunching, proteinuria, macroglossia, edema, extreme fatigue, and periorbital purpura. Some had AL amyloidosis, while others had either hereditary or wild-type transthyretin (ATTR) amyloidosis.

## Results

To test the hypotheses, a one-way ANOVA was conducted to assess the between group differences (treatment and control) on measures of active communication with patients, intention to acquire and use knowledge (of amyloidosis), and willingness to reconsider diagnoses. Results of Levene’s Test of Variances indicated homogeneity of variance between the groups ( *p* = .67) ( Adebayo, 2023a). The one-way ANOVA indicated significant differences between the groups, *F*(1, 214) = 6.72, *p* = .01, η2 = .16, power = 78. These results show statistically significant mean differences between groups. See Table 1 for descriptives.

**Table 1.** Descriptive statistics and Pearson correlations.

Dependent variable	α	M	SD	1	2	3
1 Active communication with patients	.81	6.34	.67	-	.55 *	.11 *
2 Intent to acquire & apply knowledge of amyloidosis	.78	6.59	55		-	.04 *
3 Willingness to reconsider diagnoses	.76	3.97	1.72			-

All correlations are significant at * *p* < 0.01 ( *p* is significant at 0.05), *n* = 216.

Participants in the treatment condition had significantly higher measures of active communication with patients than those in the no treatment condition *p* < 0.001. Thus, H1a was supported. Participants in the treatment condition also had significantly higher measures of intention to acquire and use knowledge about amyloidosis than those in the no treatment condition *p* < 0.001. Thus, H1b was supported. Similarly, participants in the treatment condition had significantly higher measures of willingness to reconsider a diagnosis than those in the control condition *p* = 0.001. Thus, H1c was supported. See Table 2 for descriptives.

**Table 2.** Amyloidosis Speakers Bureau (ASB) treatment and control groups’ descriptive statistics.

	Treatment group (exposed to ASB patient narrative)		Control group (not exposed to ASB patient narrative)	
**Variable**	**M**	**SD**	**M**	**SD**
Active communication with patients	6.57	.56	5.13	.59
Intent to acquire & apply knowledge of amyloidosis	6.72	.49	5.45	.47
Willingness to reconsider diagnoses	5.34	1.96	3.59	1.36

*Note*. N = 216. Treatment group (108) and control group (108).

## Discussion

This study examined the influence of patient narratives - arranged by the ASB, a nonprofit speakers bureau - on medical students’ attitudes concerning communication with patients, learning about amyloidosis, and willingness to reconsider diagnoses. Findings indicate patient narratives were effective at increasing medical students' positive attitudes toward active communication with patients, acquiring amyloidosis knowledge and using that knowledge, and willingness to reconsider diagnoses when clusters of symptoms warranted.

 These results encourage theorizing about why patient narratives had these effects, especially since research indicates the use of patient educators in medical education is under-theorized ( Gordon *et al.*, 2020). Notably, the present study looked at the impact of many patient narratives, which makes quantitative analysis possible. Forty patient narratives were delivered at 32 medical schools. These narratives had commonalities: each patient educator received the ASB’s guidelines for presenting and worked with an ASB committee member on refinements ( *Extended data,* Appendix B [ Adebayo, 2023b]). There were also differences: the types of amyloidosis experienced by patients varied as did organs and bodily systems harmed. Why then did these narratives influence medical students’ attitudes in significant ways?

There are several explanations. Slater and Rouner (2002) write that, in the case of narrative, people do not process arguments and evidence; instead, they become involved in a narrative’s characters and tension-filled moments. Thus, even though most participants reported learning about amyloidosis as part of their curriculum, the patient narratives the treatment group heard likely had a distinct influence on survey answers. 

Second, the content of these narratives was emotionally compelling. As noted by a study co-author who mentors patient speakers, they tell heartfelt stories. One patient said she went “six years” before receiving a correct diagnosis. Another “had to give up his career” and is on permanent disability because amyloidosis affected his heart. All patient educators share their pain and relief at finally receiving a correct diagnosis. In their narratives, patients did not rant about specific doctors or the medical profession. Instead, the patients' appearance before medical students suggests their perseverance in adversity and the effectiveness of their physicians. Thus, listening to patient narratives may have encouraged the treatment group to empathize with a patient educator, which influenced outcomes assessed in this study.

Third, these narratives may enact steps in the Extended Parallel Process Model (EPP; Maloney *et al.*, 2011), a well-supported persuasion theory. The EPP says four conditions encourage people to address a danger. First and second, the danger must be perceived as severe and harmful to the perceiver. Third and fourth, the recommended solution must seem effective, and the audience must feel able to enact it. The ASB patient narratives may illustrate each condition. Their stories illustrate the severity of amyloidosis, especially when not diagnosed early. These narratives also show that many doctors do not think of amyloidosis initially; therefore, participants may have felt susceptible to the harm of issuing a misdiagnosis. Third, these narratives illustrate steps that make accurate diagnosis more likely: willingness to listen to patients’ experiences, interest in learning about this disease, and humility about initial diagnoses as well as the fourth condition, evidence that medical professionals can take these steps. This analysis could be tested. One could provide a patient narrative that omits one step, such as illustrating the “solution” that is, attitudes conducive to diagnosing amyloidosis. When a message fails to establish that solutions to a severe problem exist, audiences are less apt to enact them ( Maloney *et al.*, 2011).

### Practical implications

In many curricula, didactic years precede clinical experiences where medical students encounter live patients. In contrast, some patient-centered curricula involve patient educators in the first year of medical education ( Diemers *et al.*, 2008). Exactly where in medical curricula patients’ narratives may be most effective, and why they are used, are important questions. Patient narratives concerning amyloidosis, a protein mis-folding disease, could be used to teach biochemistry, the difference between biomedical versus biopsychosocial approaches to care, or as exercises in distinguishing rare from common diseases.

Another implication concerns the benefits of a speakers bureau as compared to individual medical school faculty locating patients to speak to students. A speakers bureau with dozens of patient speakers has its advantages. Advised by faculty, the bureau recruits patient speakers, coaches them, evaluates effectiveness, manages travel or online connections, and raises funds to cover travel and incidentals. No single faculty member bears these responsibilities.

### Limitations

Like every study, this one has limitations. While the gender variation, a majority female sample, might be considered a limitation, it is consistent with the current demographic of U.S. medical schools. One might wonder if the video explaining amyloidosis harms the study design. This is unlikely given that the video focuses on the nature of the disease, its symptoms, and treatment, and says nothing about attitudes toward communication with patients nor re-thinking diagnoses. Another limitation is that this study is not designed to assess longitudinal retention of knowledge about this rare disease.

### Future research

Future work should explore the impact of specific features of patients’ narratives, such as steps that emphasize the severity of amyloidosis or steps that make diagnosis more likely. Integrating patient educators’ presentations with relevant course blocks, e.g., cardiac amyloidosis with the cardiovascular block, should also be explored. In addition, follow-up longitudinal studies should explore the outcomes in this study.

## Conclusion

 This study assessed the effectiveness of 40 ASB patient narratives sharing symptoms, diagnoses, and treatments with 216 U.S. medical students. A randomized, control group study found that listening to these presentations significantly influenced the treatment group’s willingness to (a) actively communicate with patients, (b) learn about amyloidosis, and (c) reconsider initial diagnoses when warranted. These results were explained using narrative theory. They raised questions about the conditions in which medical students most benefit from engaging with patient educators.

## Data Availability

Dataverse: Evaluating the Amyloidosis Speakers Bureau. https://doi.org/10.7910/DVN/AKXNMY ( Adebayo, 2023a) This project contains the following underlying data: Evaluating the Amyloidosis Speakers Bureau_Mar 23.tab (Data from the control and treatment groups used for analysis titled “Evaluating the Amyloidosis Speakers Bureau.”) Dataverse: Evaluating the Amyloidosis Speakers Bureau_Extended Data. https://doi.org/10.7910/DVN/7RAPLL ( Adebayo, 2023b) This project contains the following extended data: Appendix A (study measures) and Appendix B (speaker story development) titled Evaluating the Amyloidosis Speakers Bureau_ Extended Data.pdf Data are available under the terms of the Creative Commons Attribution 1.0 International license (CC0 1.0).

## References

[ref-1] Adebayo AL : Evaluating the Amyloidosis Speakers Bureau.[Dataset]. Harvard Dataverse, V1, UNF: 6:RPYfF+uhn0a1qVvZSsvAeg== [fileUNF],2023a. 10.7910/DVN/AKXNMY

[ref-2] Adebayo AL : Evaluating the Amyloidosis Speakers Bureau_Extended Data.[Dataset]. Harvard Dataverse, V1,2023b. 10.7910/DVN/7RAPLL

[ref-3] Adebayo AL Davidson Mhonde R DeNicola N : The effectiveness of narrative versus didactic information formats on pregnant women's knowledge, risk perception, self-efficacy, and information seeking related to climate change health risks. *Int J Environ Res Public Health.* 2020;17(19):6969. 10.3390/ijerph17196969 32977683 PMC7579394

[ref-4] Ajzen I : The theory of planned behaviour: Reactions and reflections. *Psychol Health.* 2011;26(9):1113–1127. 10.1080/08870446.2011.613995 21929476

[ref-5] Baker SC Watson BM Jamieson B : How do patients define satisfaction? The role of patient perceptions of their participation and health provider emotional expression. *Health Commun.* 2021;36(14):1970–1979. 10.1080/10410236.2020.1808409 32835522

[ref-6] Borah P Xiao X Lee DKL : Narrative messages, information seeking and COVID-19 vaccine intention: The moderating role of perceived behavioral control. *Am J Health Promot.* 2022;36(6):923–933. 10.1177/08901171221075019 35081757 PMC8960749

[ref-7] Borrell-Carrió F Suchman AL Epstein RM : The biopsychosocial model 25 years later: principles, practice, and scientific inquiry. *Ann Fam Med.* 2004;2(6):576–582. 10.1370/afm.245 15576544 PMC1466742

[ref-8] Bower GH Black JB Turner TJ : Scripts in memory for text. *Cogn Psychol.* 1979;11(2):177–220. 10.1016/0010-0285(79)90009-4

[ref-9] Dasari S Theis JD Vrana JA : Amyloid typing by mass spectrometry in clinical practice: a comprehensive review of 16,175 samples.In: *Mayo Clin Proc.* Elsevier,2020;95(9):1852–1864. 10.1016/j.mayocp.2020.06.029 32861330

[ref-10] Dieckmann NF Slovic P Peters EM : The use of narrative evidence and explicit likelihood by decision makers varying in numeracy. *Risk Anal.* 2009;29(10):1473–1488. 10.1111/j.1539-6924.2009.01279.x 19671102 PMC7159394

[ref-11] Diemers AD Dolmans DH Verwijnen MG : Students' opinions about the effects of preclinical patient contacts on their learning. *Adv Health Sci Educ Theory Pract.* 2008;13(5):633–647. 10.1007/s10459-007-9070-6 17629786

[ref-12] Dispenzieri A Merlini G : Future perspectives. *Hematol Oncol Clin North Am.* 2020;34(6):1205–1214. 10.1016/j.hoc.2020.08.009 33099434 PMC7576440

[ref-13] Drewniak D Glässel A Hodel M : Risks and benefits of web-based patient narratives: Systematic review. *J Med Internet Res.* 2020;22(3):e15772. 10.2196/15772 32213468 PMC7146251

[ref-14] Edwards PC Graham J Oling R : The patient educator presentation in dental education: Reinforcing the importance of learning about rare conditions. *J Dent Educ.* 2016;80(5):533–541. 10.1002/j.0022-0337.2016.80.5.tb06113.x 27139204

[ref-15] Engel GL : The need for a new medical model: A challenge for biomedicine. *Science.* 1977;196(4286):129–136. 10.1126/science.847460 847460

[ref-16] Fetterman AK Curtis S Carre J : On the willingness to admit wrongness: Validation of a new measure and an exploration of its correlates. *Pers Individ Dif.* 2019;138:193–202. 10.1016/j.paid.2018.10.002

[ref-17] Fisher WR : Narration as a human communication paradigm: The case of public moral argument. *Commun Monogr.* 1984;51(1):1–22. 10.1080/03637758409390180

[ref-18] Gigerenzer G Gaissmaier W Kurz-Milcke E : Helping doctors and patients make sense of health statistics. *Psychol Sci Public Interest.* 2007;8(2):53–96. 10.1111/j.1539-6053.2008.00033.x 26161749

[ref-19] Gillmore JD Gane E Taubel J : CRISPR-Cas9 in vivo gene editing for transthyretin amyloidosis. *N Engl J Med.* 2021;385(6):493–502. 10.1056/NEJMoa2107454 34215024

[ref-20] Gordon M Gupta S Thornton D : Patient/service user involvement in medical education: A best evidence medical education (BEME) systematic review: BEME Guide No. 58. *Med Teach.* 2020;42(1):4–16. 10.1080/0142159X.2019.1652731 31518544

[ref-21] Green MC : Narratives and cancer communication. *J Commun.* 2006;56(s1):S163–S183. 10.1111/j.1460-2466.2006.00288.x

[ref-22] Grogan M Dispenzieri A Gertz MA : Light-chain cardiac amyloidosis: strategies to promote early diagnosis and cardiac response. *Heart.* 2017;103(14):1065–1072. 10.1136/heartjnl-2016-310704 28456755 PMC5566095

[ref-23] Hinyard LJ Kreuter MW : Using narrative communication as a tool for health behavior change: a conceptual, theoretical, and empirical overview. *Health Educ Behav.* 2007;34(5):777–792. 10.1177/1090198106291963 17200094

[ref-24] Keshet Y Schiff E Samuels N : Giving voice to cancer patients: assessing non‐specific effects of an integrative oncology therapeutic program via short patient narratives. *Psychooncology.* 2015;24(2):169–174. 10.1002/pon.3621 25043932

[ref-25] Langer N Ribarich M : Using narratives in healthcare communication. *Educ Gerontol.* 2008;35(1):55–62. 10.1080/03601270802388930

[ref-26] Lousada I Comenzo RL Landau H : Light chain amyloidosis: Patient experience survey from the Amyloidosis Research Consortium. *Adv Ther.* 2015;32(10):920–928. 10.1007/s12325-015-0250-0 26498944 PMC4635176

[ref-27] Maloney EK Lapinski MK Witte K : Fear appeals and persuasion: A review and update of the Extended Parallel Process Model. *Soc Personal Psychol Compass.* 2011;5(4):206–219. 10.1111/j.1751-9004.2011.00341.x

[ref-28] McCausland KL White MK Guthrie SD : Light chain (AL) amyloidosis: the journey to diagnosis. *Patient.* 2018;11(2):207–216. 10.1007/s40271-017-0273-5 28808991 PMC5845075

[ref-29] Petty RE Cacioppo JT : The Elaboration Likelihood Model of Persuasion. *Adv Exp Soc Psychol.* 1986;19:123–205. 10.1016/S0065-2601(08)60214-2

[ref-30] Shaffer VA Focella ES Hathaway A : On the usefulness of narratives: An interdisciplinary review and theoretical model. *Ann Behav Med.* 2018;52(5):429–442. 10.1093/abm/kax008 29684135 PMC6369912

[ref-31] Slater MD Rouner D : Entertainment-education and elaboration likelihood: Understanding the processing of narrative persuasion. *Communication Theory.* 2002;12(2):173–191. 10.1111/j.1468-2885.2002.tb00265.x

[ref-32] Snow R Crocker J Talbot K : Does hearing the patient perspective improve consultation skills in examinations? An exploratory randomized controlled trial in medical undergraduate education. *Med Teach.* 2016;38(12):1229–1235. 10.1080/0142159X.2016.1210109 27573531

[ref-33] Todres L Galvin KT Holloway I : The humanization of healthcare: A value framework for qualitative research. *Int J Qual Stud Health Well-being.* 2009;4(2):68–77. 10.1080/17482620802646204

