# Supplementary material for: Evaluating the Amyloidosis Speakers Bureau: the influence of amyloidosis patients’ narratives on medical students’ knowledge, attitudes, and behavioral intent
Source: MedEdPublish (2016). 2023 Jun 28;13:32. [Version 1] doi: 10.12688/mep.19631.1 (PMC10518845; doi:10.12688/mep.19631.1)
Supplement: Peer review history

# Reviewer report: Reviewer response for version 1

**DOI:** 10.21956/mep.21030.r34550

**Published:** 2023-09-14

**Authors:** Federico Perfetto

**Affiliations:** 1 Careggi University Hospital, Florence, Italy

Diagnosing and treating amyloidosis still represents an important challenge for the physicians both due to the rarity of this pathology and the often non-specific clinical presentation with non-specific signs and symptoms. This leads to a diagnostic delay in at least 30% of patients with serious consequences in terms of mortality and morbidity.

 Students' knowledge of amyloidosis is very often based only on a few memories acquired during his curricula and due to its rarity, almost never on living patients experiences. Furthermore, the concept that amyloidosis is an incurable disease still remains, which leads to a nihilistic attitude towards it.

 The possibility of hearing the narration of a specific illness directly from patients represent a fundamental aspect of the knowledge of the pathology which may allows to broaden our understanding of how this disease manifests itself and evolves. This is even more true in a rare and complex disease such as systemic amyloidosis. The possibility of listening to the tortuous clinical-diagnostic pathways of these patients, their ups and downs, the erroneous diagnoses received, in a training phase of the student will improve, as this study clearly demonstrates, the attitude of medical students regarding communication with patients, their learning about amyloidosis, and willingness to reconsider diagnoses The protocol is very simple and well illustrated by the authors and the experiment was conducted using two groups of students employing only one post-test measurement. Furthermore, to reduce sensitization of study participants, the possibility of conducting pre-test measures was eliminated. Therefore, the group that was not exposed to the patient's story was considered as the control group.

 Comparing the treatment group with the control the patient narratives showed effective at increasing medical students' positive attitudes toward active communication with patients, acquiring amyloidosis knowledge and using that knowledge, and willingness to reconsider diagnoses when clusters of symptoms warranted.

 The article is well balanced the references are adequate and appropriate and the results and discussion are well highlighted and commented.

Have any limitations of the research been acknowledged?

No

Is the work clearly and accurately presented and does it cite the current literature?

Yes

If applicable, is the statistical analysis and its interpretation appropriate?

Yes

Are all the source data underlying the results available to ensure full reproducibility?

Yes

Is the study design appropriate and is the work technically sound?

Yes

Are the conclusions drawn adequately supported by the results?

Yes

Are sufficient details of methods and analysis provided to allow replication by others?

Yes

Reviewer Expertise:

Internal Medicine

I confirm that I have read this submission and believe that I have an appropriate level of expertise to confirm that it is of an acceptable scientific standard.

# Reviewer report: Reviewer response for version 1

**DOI:** 10.21956/mep.21030.r34004

**Published:** 2023-07-18

**Authors:** Morie Gertz

**Affiliations:** 1 Division of Hematology, Mayo Clinic, Rochester, MN, USA

The major gap in improving outcomes for patients with amyloidosis is an early diagnosis. There remains a major Education gap in the recognition of this rare disease given its lack of specific signs and symptoms leading to the diagnosis. This trial explores methods to close this gap. Students are exposed to a patient with this disease hearing 1st hand the symptoms leading to diagnosis any of the potential delays in coming to the correct diagnosis. This is followed by a randomized post test tool that compared treatment and control in terms of knowledge of the presenting symptoms of the disease and methods in which more effective communication with patients might be undertaken. It is well written and well referenced all data is available for review and the technique is adaptable for a whole host of rare disorders and can be copied.

Have any limitations of the research been acknowledged?

Yes

Is the work clearly and accurately presented and does it cite the current literature?

Yes

If applicable, is the statistical analysis and its interpretation appropriate?

Yes

Are all the source data underlying the results available to ensure full reproducibility?

Yes

Is the study design appropriate and is the work technically sound?

Yes

Are the conclusions drawn adequately supported by the results?

Yes

Are sufficient details of methods and analysis provided to allow replication by others?

Yes

Reviewer Expertise:

Clinical hematologist with expertise in the diagnosis of amyloidosis

I confirm that I have read this submission and believe that I have an appropriate level of expertise to confirm that it is of an acceptable scientific standard.
